# Serum biomarkers for the early diagnosis of TIA: The MIND-TIA study protocol

**DOI:** 10.1186/s12883-015-0388-z

**Published:** 2015-07-28

**Authors:** L. Servaas Dolmans, Frans H. Rutten, Marie-Louise EL Bartelink, Gerdien Seppenwoolde, Sanne van Delft, L. Jaap Kappelle, Arno W. Hoes

**Affiliations:** Julius Center for Health Sciences and Primary Care, University Medical Center Utrecht, Utrecht, The Netherlands; Saltro Diagnostic Center for Primary Care, Utrecht, The Netherlands; Department of Neurology, University Medical Center Utrecht, Utrecht, The Netherlands

**Keywords:** TIA, Minor stroke, Diagnosis, Biomarkers

## Abstract

**Background:**

A Transient Ischaemic Attack (TIA) bears a high risk of a subsequent ischaemic stroke. Adequate diagnosis of a TIA should be followed immediately by the start of appropriate preventive therapy, including antiplatelets. The diagnosis of a TIA based on symptoms and signs only is notoriously difficult and biomarkers of brain ischaemia might improve the recognition, and target management and prognosis of TIA patients. Our aim is to quantify the added diagnostic value of serum biomarkers of brain ischaemia in patients suspected of TIA.

**Methods/design:**

Study design: a cross-sectional diagnostic accuracy study with an additional six month follow-up period.

Study population: 350 patients suspected of TIA in the primary care setting.

Patients suspected of a TIA will be recruited by at least 200 general practitioners (GPs) in the catchment area of seven TIA outpatient clinics willing to participate in the study. In all patients a blood sample will be drawn as soon as possible after the patient has contacted the GP, but at least within 72 h after onset of symptoms. Participants will be referred by the GP to the regional TIA outpatient clinic for additional investigations, including brain imaging. The ‘definite’ diagnosis (reference standard) will be made by a panel consisting of three experienced neurologists who will use all available diagnostic information and the clinical information obtained during the outpatient clinic assessment, and a six month follow-up period. The diagnostic accuracy, and value in addition to signs and symptoms of candidate serum biomarkers will be assessed in terms of discrimination with C statistics, and calibration with plots.

We aim to include 350 suspected cases, with 250 patients with indeed definite TIA (or minor stroke) according to the panel.

**Discussion:**

We hope to find novel biomarkers that will enable a rapid and accurate diagnosis of TIA. This would largely improve the management and prognosis of such patients.

**Trial registration:**

ClinicalTrials.gov Identifier NCT01954329

## Background

Cerebrovascular disease is the second leading cause of death in Europe and a leading cause of long- term disability [1]. A Transient Ischaemic Attack (TIA) by definition does not result in permanent damage of brain tissue [2], but the risk of a subsequent ischaemic stroke is substantial, especially within the first 2 weeks. Moreover, high-resolution MRI of the brain showed that many TIAs should be regarded as a minor stroke by revealing small ischaemic lesions. About 5 % of TIA patients will have a major stroke within 48 h, and another 5 % within 3 months [3, 4]. Urgent treatment of TIA patients with preventive treatment reduced the risk of stroke within 3 months by up to 80 % in a non-experimental study. An absolute reduction in stroke incidence from 10.3 % to 2.1 % was observed with implementing urgent diagnostic assessment followed immediately by adequate treatment including antiplatelets, when compared to a historical cohort in the same hospital in the UK [5]. Therefore, early recognition of TIA is of great importance. Recent studies underline the fact that TIA is a medical emergency [6] and adequate antithrombotic treatment reduces disability and health care related costs [7]. However, a rapid start of treatment may be hampered by patient and physician delay, waiting time involved in referral to the TIA outpatient clinic, and difficulties in establishing the correct diagnosis.

Diagnosing TIA is notoriously difficult for physicians. It is primarily based on history taking, since symptoms and signs often have resolved by the time the patient consults a physician, typically a general practitioner (GP). Symptoms may be inadequately observed by the patient or eyewitnesses, and the history can be distorted by difficulties in recalling the event. It is often difficult for lay persons to narrate the experienced symptoms. Moreover, TIAs can have a non-specific presentation and the differential diagnosis is broad. Particularly TIAs that originate from the vertebrobasilar artery system are difficult to recognise and hard to distinguish from other more benign causes of symptoms like dizziness. Neuroimaging techniques such as CT and MRI scanning of the brain are not performed to confirm the diagnosis of TIA, but to rule out other cerebral diagnoses, including cerebral haemorrhage [8]. In patients referred to a TIA service by the GP in Western Europe, the diagnosis TIA is confirmed by the neurologist in about 70 % of cases [9–11]. Even among experienced neurologists, however, there is substantial interobserver disagreement in TIA diagnosis, with Cohen’s kappa statistics varying from 0.65 to 0.78 [11]*.*

A possible solution to the diagnostic difficulties in TIA would be a serum biomarker that can reliably detect (transient) brain ischaemia in an early phase after symptom onset. This would enable a more accurate diagnosis within a shorter time frame. Especially in the primary care setting, but also in the emergency department, such a test would be very useful when available, preferably as a point of care (POC) test.

Biomarkers could also provide valuable prognostic information. Some markers have already shown to be helpful in predicting the risk of an ischaemic stroke within 2 weeks. This could be useful to guide rapid referral to a neurologist and early initiation of intensive treatment of risk factors, including anticoagulation in patients with atrial fibrillation, and carotid endarterectomy in cases with clinically relevant narrowing of the carotid artery.

There is a growing list of biomarkers associated with different components of the ischaemic cascade in the brain. To be useful in the diagnosis of TIA, a biomarker should be sensitive to early ischaemia and specific for the brain [12–14]. The biomarker should preferably be released in blood immediately after the ischaemic event and remain detectable for several days because it is then also applicable to patients who have a substantial patient delay. Based on a systematic review of the literature we selected seven biomarkers that potentially meet these criteria and showed to have diagnostic potential (table 1). The selection of markers is not definite and will be updated prior to our actual measurements. Previous biomarker studies in the field of cerebral ischemia up to now mainly focused on major stroke and showed methodological limitations which we want to overcome in the present study. To our knowledge, this study, Markers in the Diagnosis of TIA (MIND-TIA), is the first to evaluate the value of serum biomarkers in patients suspected of TIA in addition to history taking.

**Table 1:** Main characteristics of potential diagnostic biomarkers for TIA

Biomarker (abbreviation)	Full name	Main biological action	Main study reference
B-FABP	Brain-type fatty acid binding protein	Protein involved in the intracellular transport and oxidation of fatty acids, and membrane lipid trafficking, expressed in glial cells	Wunderlich, et al. [20]
H-FABP	Heart-type fatty acid binding protein	Protein involved in the intracellular transport and oxidation of fatty acids, and membrane lipid trafficking, expressed in myocardium but also in neuronal cell bodies in the central nervous system	Wunderlich, et al. [20]
PARK7	Parkinson protein 7	RNA binding protein regulatory subunit, protects neurons against oxidative stress and cell death	Allard, et al. [21]
NDKA	Nucleoside diphosphate kinase A	Enzyme catalysing transfer of phosphate groups between nucleoside tri-phosphates and nucleoside diphosphates (e.g. ATP to GDP), expressed in neurons	Allard, et al. [21]
UFDP	Ubiquitin fusion degradation protein 1	Enzyme in the pathway for degrading ubiquitin-protein conjugates, involved in protein degradation in cell damage	Allard, et al. [22]
NR2A/2B	N-Methyl-D-aspartate (NMDA) receptor subunits	Product of the proteolytic degradation of NMDA receptors (part of the ischaemic cascade in the brain)	Dambinova, et al. [23]
NR2A/2B Ab	N-Methyl-D-aspartate (NMDA) receptor antibodies	Antibodies to NMDA receptor fragments	Weismann, et al. [24]

This paper presents the MIND-TIA study protocol.

### Primary objective

- ▪To assess the added diagnostic value of serum biomarkers beyond symptoms and signs in patients suspected of TIA.

### Secondary objective

- ▪To assess the short-term prognostic value of serum biomarkers in patients with TIA.

## Methods/design

### Study design

A cross-sectional diagnostic accuracy study with an additional 6 months follow-up period. Participants are patients suspected of a TIA by their GP. In all participants we will perform a biomarker assessment (index test) and the ‘definite’ diagnosis of TIA will be determined by an expert panel diagnosis (reference standard). A panel of three neurologists will evaluate all available diagnostic information, including imaging of the brain and additional ‘diagnostic’ information that became available in the 6 months of follow-up after the ‘event’ (so called delayed verification).

### Study population and setting

The study population will consist of patients suspected of a new (not necessarily first) possible TIA by the GP. Patients are recruited within 72 h after symptom onset and either directly after their GP consultation or at the time they visit the TIA outpatient clinic after referral by the GP. We will use the following inclusion and exclusion criteria:

#### Inclusion criteria

- ▪Age 18 years and older.
- ▪A new episode of symptoms or signs suspected of TIA for which the GP considers referral to the TIA outpatient clinic for further investigations to confirm or exclude TIA, or for additional treatment.
- ▪A blood sample can be collected within 72 h after onset of symptoms.
- ▪Written informed consent.

#### Exclusion criteria

- ▪Patients that still have active symptoms or signs at the time of recruitment (i.e. during consultation of the GP), and thus are suspected of an ongoing stroke.
- ▪If valid history taking is impossible because of severe cognitive impairment or insufficient knowledge of the Dutch language.
- ▪Patients with a life expectancy of less than 6 months.

We aim to include 350 patients suspected of TIA, targeting at 250 patients who show to have a definite diagnosis of TIA according to the panel. Following the sample size calculation below, we need at least 200 recruiting GPs in the catchment area of four to five hospitals with a TIA outpatient clinic to complete inclusion within 2 years. Geographically, the study region in the centre of the Netherlands contains around 900 GPs and seven TIA outpatient clinics.

### Recruitment and consent

Patients suspected of TIA will be recruited (not included) by the participating GP or at the time they visit the TIA outpatient clinic after GP referral. The GP or TIA outpatient clinic personnel will ask if the patient agrees to be contacted by the researcher by telephone to explain the study and discuss possible participation.

The researcher will check whether the patient is eligible and still willing to participate. If so, a home visit by a research nurse is arranged and after the patient has signed informed consent, he/she is included in the study and further study procedures are initiated.

### Outcome measures

#### Diagnosis of TIA

Main study endpoint is the diagnosis of TIA (or minor stroke). The expert panel classifies subjects into i) TIA, ii) minor (or even major) ischaemic stroke, or iii) other diagnoses (haemorrhagic cerebrovascular disease or non-cerebrovascular disease). In the analysis we will use a composite endpoint of TIA and minor ischaemic stroke.

#### Endpoints after six months of follow-up

In order to improve the final diagnosis by the panel and to evaluate the short-term prognostic value of the set of biomarkers we will assess the occurrence of (ischaemic) cerebrovascular events and (ischaemic) cardiovascular events, and mortality during six months of follow-up.

The primary prognostic endpoint will be a composite endpoint of ischaemic stroke and (all- cause) mortality within the 6 months follow-up period. This outcome is most often used in prognostic studies concerning cerebrovascular disease.

Secondary endpoint(s) are:

- ▪The composite of recurrent TIA or ischaemic stroke, all-cause mortality, and high-risk stroke mechanism requiring specific early intervention (the latter defined as the presence of a treatment-emergent mechanism for which a specific therapy other than antiplatelet therapy is indicated, i.e. stenosis of the carotid artery necessitating carotid endarterectomy or a cardioembolic source warranting anticoagulation)

### Study procedures

Our study design aims to mimic the routine diagnostic routing of TIA as much as possible, as is common for diagnostic studies. For study reasons, a research nurse will visit the participant at home to draw an extra blood sample as soon as possible after inclusion for assessment of the biomarkers. After blood sampling participants complete a health-related questionnaire and the research nurse will fill out a standardised case record form (CRF) with items on history taking.

Following routine care, the GP will refer participants to the regional TIA outpatient clinic for further investigations and (additional) treatment. The neurologist will determine, in accordance with common practice, which additional tests should be performed. In the Netherlands the diagnostic evaluation at TIA outpatient clinics is organized according to national guidelines and is similar among hospitals. Brain imaging is performed in all patients suspected of TIA/minor stroke at the TIA outpatient clinics, nearly always CT scanning but increasingly next to this also MRI.

We will collect data on the following tests/assessments:

1. The findings during medical history taking and the physical examination by the GP. This will be done retrospectively when patients are included.
2. Venous blood sampling (20 ml of blood) for assessment of a set of biomarkers blinded to other results.
3. Case record form (CRF) with standardised history taking, completed by the research nurse and including a narrative account back-upped on tape of the signs and symptoms by the patient.
4. Findings during the clinical assessment of the neurologist at the TIA outpatient clinic, including electrocardiography, and if performed, carotid duplex scan and CT (or MRI) of the brain.
5. Follow-up assessment of the six months following the event by scrutinising the electronical medical files of the participating GPs, with collection of data on all endpoints.

### Biomarker assessment

We will assess the levels of a set of biomarkers in a sample of blood taken within 72 h after onset of symptoms.

We will collect 20 ml of venous blood by venepuncture. The whole blood samples will be transported immediately to Saltro Diagnostic Center (an accredited primary care diagnostic facility in the Utrecht region) in a Cool Transport container. Pre-analytical processing will be performed within 3 h after collection. Serum will be separated by centrifugation at 2500 g for 10 min. The serum samples will then be stored in 0.5 ml aliquots at −80 °C and transported to the University Medical Center of Utrecht Biobank for long-term storage.

We plan to assess the following biomarkers by sandwich enzyme-linked immunosorbent assay (ELISA) procedures: B-FABP, H-FABP, PARK-7, NDKA, UFDP, NR2 and NR2Ab. These measurements will be performed at the end of the study in one single batch, and blinded to other results and outcomes.

The surplus of serum and a buffy coat will be stored to facilitate future (biomarker) investigations in (suspected) TIA patients.

### Panel diagnosis

An expert panel consisting of three neurologists will evaluate paper-based summaries of all case record forms (including medical history, initial signs and symptoms, the patient’s own narrative account of symptoms), reports of the neurologist, radiological imaging reports on brain imaging and carotid artery function, and six months of follow-up.

The panel will classify whether the patient has had a TIA, a minor (or even major) ischaemic stroke, or any other diagnosis. They will follow the definitions from the scientific statement of the American Heart Association ‘Definition and evaluation of TIA’ (2009) [2]. Within the group of TIA or minor strokes, the panel will also determine the aetiology of the ischaemic event, i.e. cardioembolic, large artery atherosclerotic, lacunar, other or undetermined aetiology. The panel judgement is made without knowledge of the biomarker values.

Every panel member will first assess the cases individually. Cases in which the panel members disagree will be discussed in a plenary meeting and a final decision will be made by voting, with the majority of votes counting. Panel meetings will be led by the researcher, who is responsible for providing all necessary data, but who will not participate in the consensus discussions.

Reproducibility of the panel diagnosis will be evaluated by calculating the inter-rater agreement with kappa statistics and by assessment of the reproducibility of the plenary decision process by reassessing a sample of around 10 % of the patients.

### Sample size calculation

Our sample size calculation is based on the primary research question to be able to answer whether any of the biomarkers has added diagnostic value beyond the clinical assessment. We applied Harrell’s rule of thumb [15] that may be used for power calculations in diagnostic and prognostic studies. This rule states that for every determinant considered for multivariate logistic regression analysis at least ten subjects are needed in the smallest category of the outcome variable.

On a TIA service the diagnosis of TIA is confirmed by the neurologist in around 70 % of patients referred by the GP [11, 16]. About 30 % of patients will be diagnosed as non-TIA (the latter being the smallest outcome category).

Following these proportions and because we evaluate up to 10 potential diagnostic determinants, 100 non-TIA patients are required. This means that we need a total of (100 x (1/0.3)=) 333 patients suspected of TIA. To be on the safe side and to allow for some ‘loss to follow-up’ and missing of essential endpoints, we aim to include 350 patients.

In case the proportion of non-TIA patients is higher than 30 %, less than 350 suspected TIA will suffice: we will stop inclusion after a total of 100 non-TIA patients have been verified by the panel and included in the study.

We realize that the power is insufficient for answering the secondary research question on prognosis based on an expected incidence of 20–30 follow-up events in six months. At the best, two or three predictors could be evaluated in multivariable regression analysis. The results on prognosis will therefore be hypothesis generating rather than hypothesis testing.

### Data analysis

#### Diagnostic study

The final TIA diagnosis will be presented as frequencies of the composite of TIA or minor stroke versus other diagnoses. The parameters of routine clinical assessment (symptoms and signs) of the GP and the mean biomarker values will be presented for subjects with a TIA/minor stroke and subjects with other diagnoses. First, the positive and negative predictive value and sensitivity and specificity will be assessed as test characteristics of all diagnostic tests/biomarkers.

Multiple logistic regression analyses with and without biomarker test results will be performed after multiple imputation of missings, to quantify the diagnostic accuracy of the routine clinical assessment by the GP (first model) and the improvement of diagnostic accuracy by adding biomarker assessment to this clinical assessment (additional models). Overall diagnostic accuracy of the models (after adjustment for over-optimism using bootstrapping techniques) will be quantified by assessing and comparing their calibration (applying the Hosmer-Lemeshow test) and discrimination (using ROC area or C-statistics) and classification across various probability cut-offs (e.g. using the integrated discrimination index and the net reclassification improvement).

#### Prognostic study

The nowadays advocated prognostic ABCD2-score (Age ≥ 60, Blood pressure ≥140/90 mmHg, Clinical features, Duration of symptoms, Diabetes) will be assessed in each subject. Because we expect a low number of short-term events, we will only explore the predictive ability of the ABCD2-score, the biomarkers, and ABCD2 plus biomarkers(s).

### Regulation statement

This study is conducted according to the principles of the current version of the declaration of Helsinki and in accordance with the Dutch law on Medical Research Involving Human Subjects Act (WMO).

### Ethics committee approval

Ethical approval was given by the Medical Research Ethics Committee of the University Medical Center of Utrecht, the Netherlands, on September 5^th^ 2013.

## Discussion

In the MIND-TIA study we hope to find novel biomarkers that improve the accuracy of the GP’s diagnosis in patients suspected of TIA. This would result in a more appropriate assessment of patients suspected of TIA and timely treatment, and thus improved prognosis of TIA patients.

The study will follow clinical practice. This will help future implementation of the results in daily practice. Early blood sampling is necessary because some (potential) markers of brain ischaemia can no longer be detected 72 h after the onset of symptoms and because early treatment of TIA is essential to optimize prognosis. Diagnostic research should involve patients *suspected* of a certain disease, and results of tests should be considered *in addition* to already available test results from the clinical assessment, thus following the natural diagnostic hierarchy. In our study we thus aim to evaluate the *added* value of biomarkers beyond the clinical assessment of the GP.

The success of our study depends on the shared effort of a large number of GPs. To improve participation, we facilitate the inclusion process by involving trained research nurses who do the home visits including the informed consent procedure.

In diagnostic accuracy studies creating a valid reference standard is of a major concern. Specifically in the case of TIA, an acceptable reference standard diagnosis is challenging. In lack of a single reference test or the possibility of a composite reference standard, panel diagnosis is the only acceptable method for obtaining a final TIA diagnosis [17, 18]. Our panel will evaluate diagnostic information collected through various sources (GP, research nurse and neurologist), including a clinical follow-up period of six months [19]. We aim for transparent reporting of the decision making process of the panel, including assessing its reproducibility.

## References

[1] Nichols M, Townsend N, Luengo-Fernandez R, Leal J, Gray A, Scarborough P (2012). European Heart Network.

[2] Easton JD, Saver JL, Albers GW, Alberts MJ, Chaturvedi S, Feldmann E (2009). Definition and evaluation of transient ischemic attack: a scientific statement for healthcare professionals from the American Heart Association/American Stroke Association Stroke Council; Council on Cardiovascular Surgery and Anesthesia; Council on Cardiovascular Radiology and Intervention; Council on Cardiovascular Nursing; and the Interdisciplinary Council on Peripheral Vascular Disease. Stroke.

[3] Giles MF, Rothwell PM (2007). Risk of stroke early after transient ischaemic attack: a systematic review and meta-analysis. Lancet Neurol.

[4] Wu CM, McLaughlin K, Lorenzetti DL, Hill MD, Manns BJ, Ghali WA (2007). Early risk of stroke after transient ischemic attack: a systematic review and meta-analysis. Arch Intern Med.

[5] Rothwell PM, Giles MF, Chandratheva A, Marquardt L, Geraghty O, Redgrave JN (2007). Effect of urgent treatment of transient ischaemic attack and minor stroke on early recurrent stroke (EXPRESS study): a prospective population-based sequential comparison. Lancet.

[6] Kappelle LJ (2007). A transient ischaemic attack (TIA) is an emergency. Ned Tijdschr Geneeskd.

[7] Luengo-Fernandez R, Gray AM, Rothwell PM (2009). Effect of urgent treatment for transient ischaemic attack and minor stroke on disability and hospital costs (EXPRESS study): a prospective population-based sequential comparison. Lancet Neurol.

[8] Chalela JA, Kidwell CS, Nentwich LM, Luby M, Butman JA, Demchuk M (2007). Magnetic resonance imaging and computed tomography in emergency assessment of patients with suspected acute stroke: a prospective comparison. Lancet.

[9] Amarenco P, Labreuche J, Lavallee PC (2012). Patients with transient ischemic attack with ABCD2 < 4 can have similar 90-day stroke risk as patients with transient ischemic attack with ABCD2 >/=4. Stroke.

[10] Amort M, Fluri F, Schafer J, Weisskopf F, Katan M, Burow A (2011). Transient ischemic attack versus transient ischemic attack mimics: frequency, clinical characteristics and outcome. Cerebrovasc Dis.

[11] Fonseca AC, Canhao P (2011). Diagnostic difficulties in the classification of transient neurological attacks. Eur J Neurol.

[12] Jensen MB, Chacon MR, Sattin JA, Aleu A, Lyden PD (2008). The promise and potential pitfalls of serum biomarkers for ischemic stroke and transient ischemic attack. Neurologist.

[13] Kernagis DN, Laskowitz DT (2012). Evolving role of biomarkers in acute cerebrovascular disease. Ann Neurol.

[14] Maas MB, Furie KL (2009). Molecular biomarkers in stroke diagnosis and prognosis. Biomark Med.

[15] Harrell FE, Lee KL, Califf RM, Pryor DB, Rosati RA (1984). Regression modelling strategies for improved prognostic prediction. Stat Med.

[16] Lavellee PC, Mesequer E, Abboud H, Cabrejo L, Olivot JM, Simon O (2007). A transient ischaemic attack clinic with round-the-clock access (SOS-TIA): feasibility and effects. Lancet Neurol.

[17] Castle J, Mlynash M, Lee K, Caulfield AF, Wolford C, Kemp S (2010). Agreement regarding diagnosis of transient ischemic attack fairly low among stroke-trained neurologists. Stroke.

[18] Moons KG, Grobbee DE (2002). When should we remain blind and when should our eyes remain open in diagnostic studies?. J Clin Epidemiol.

[19] Reitsma JB, Rutjes AW, Khan KS, Coomarasamy A, Bossuyt PM (2009). A review of solutions for diagnostic accuracy studies with an imperfect or missing reference standard. J Clin Epidemiol.

[20] Wunderlich MT, Hanhoff T, Goertler M, Spener F, Glatz J, Wallesch CW (2005). Release of brain-type and heart-type fatty acid-binding proteins in serum after acute ischaemic stroke. J Neurol.

[21] Allard L, Burkhard PR, Lescuyer P, Burgess JA, Walter N, Hochstrasser DF (2005). PARK7 and nucleoside diphosphate kinase A as plasma markers for the early diagnosis of stroke. Clin Chem.

[22] Allard L, Turck N, Burkhard PR, Walter N, Rosell A, Gex-Fabry M (2007). Ubiquitin fusion degradation protein 1 as a blood marker for the early diagnosis of ischemic stroke. Biomark Insights.

[23] Dambinova SA, Bettermann K, Glynn T, Tews M, Olson D, Weissman DJ (2012). Diagnostic potential of the NMDA receptor peptide assay for acute ischemic stroke. PLoS One.

[24] Weissman JD, Khunteev GA, Heath R, Dambinova SA (2011). NR2 antibodies: risk assessment of transient ischemic attack (TIA)/stroke in patients with history of isolated and multiple cerebrovascular events. J Neurol Sci.

